# Genome-wide association study identifies two new susceptibility loci for colorectal cancer at 5q23.3 and 17q12 in Han Chinese

**DOI:** 10.18632/oncotarget.5530

**Published:** 2015-10-26

**Authors:** Kewei Jiang, Yimin Sun, Cheng Wang, Jiafu Ji, Yaoping Li, Yingjiang Ye, Liang Lv, Yong Guo, Sutang Guo, Hai Li, Lianhai Zhang, Yanbing Zhou, Bo Jiang, Yonghong Ren, Youchun Xu, Xiongfei Yang, Hongxia Liu, Yirui Wang, Zhanlong Shen, Wenyan Qin, Peng Guo, Yuyang Jiang, Zhibin Hu, Hongbing Shen, Jing Cheng, Yinxue Yang, Shan Wang

**Affiliations:** ^1^ Department of General Surgery, Laboratory of Surgical Oncology, Peking University People's Hospital, Beijing 100044, China; ^2^ Health Science Research Institute, Capital Bio Corporation, Beijing 102206, China; ^3^ National Engineering Research Center for Beijing Biochip Technology, Beijing 102206, China; ^4^ The State Key Laboratory Breeding Base-Shenzhen Key Laboratory of Chemical Biology, The Graduate School at Shenzhen, Tsinghua University, Shenzhen 518055, China; ^5^ Section of Clinical Epidemiology, Jiangsu Key Laboratory of Cancer Biomarkers, Prevention and Treatment, Cancer Center, Nanjing Medical University, Nanjing 211166, China; ^6^ Department of Epidemiology and Biostatistics and Ministry of Education (MOE) Key Lab for Modern Toxicology, School of Public Health, Nanjing Medical University, Nanjing 211166, China; ^7^ State Key Laboratory of Reproductive Medicine, Nanjing Medical University, Nanjing 210029, China; ^8^ Department of Surgery, Ministry of Education Key Lab of Carcinogenesis and Translational Research, Peking University Cancer Hospital & Institute, Beijing 100142, China; ^9^ Department of Colorectal Surgery, Shanxi Cancer Hospital and Institute, Affiliated Cancer Hospital of Shanxi Medical University, Taiyuan 030001, China; ^10^ Shanxi Branch Center, National Engineering Research Center for Beijing Biochip Technology, Taiyuan, China; ^11^ Department of General Surgery, Affiliated Hospital of Qingdao University Medical College, Qingdao 266003, China; ^12^ Department of Biomedical Engineering, Medical Systems Biology Research Center, Tsinghua University School of Medicine, Beijing 100084, China; ^13^ Department of Molecular Biology, Shanxi Cancer Hospital and Institute, Affiliated Cancer Hospital of Shanxi Medical University, Taiyuan 030001, China; ^14^ Department of Anal-Colorectal Surgery, General Hospital of Ningxia Medical University, Yinchuan 750004, China; ^15^ The Anorectal Department, Gansu Provincial People's Hospital, Lanzhou 730000, China; ^16^ The State Key Laboratory of Biomembrane and Membrane Biotechnology, Tsinghua University, Beijing 100084, China; ^17^ Ningxia Branch Center, National Engineering Research Center for Beijing Biochip Technology, Yinchuan 750004, China

**Keywords:** GWAS, colorectal cancer, association

## Abstract

Genome-wide association studies (GWAS) have reported a number of loci harboring common variants that influence risk of colorectal cancer (CRC) in European descent. But all the SNPs identified explained a small fraction of total heritability. To identify more genetic factors that modify the risk of CRC, especially Chinese Han specific, we conducted a three-stage GWAS including a screening stage (932 CRC cases and 966 controls) and two independent validations (Stage 2: 1,759 CRC cases and 1,875 controls; Stage 3: 943 CRC cases and 1,838 controls). In the combined analyses, we discovered two novel loci associated with CRC: rs12522693 at 5q23.3 (CDC42SE2-CHSY3, OR = 1.31, P = 2.08 × 10^−8^) and rs17836917 at 17q12 (ASIC2-CCL2, OR = 0.75, P = 4.55 × 10^−8^). Additionally, we confirmed two previously reported risk loci, rs6983267 at 8q24.21 (OR = 1.17, P = 7.17 × 10^−7^) and rs10795668 at 10p14 (OR = 0.86, P = 2.96 × 10^−6^) in our cohorts. These results bring further insights into the CRC susceptibility and advance our understanding on etiology of CRC.

## INTRODUCTION

Colorectal cancer (CRC) is the third most commonly diagnosed cancer in males and the second in females worldwide [1]. To date, genome-wide association studies (GWAS) have identified more than twenty loci associated with CRC risk in European descent [2–13] and more than ten loci in East Asian descent [14, 15], which partly elucidated the genetic basis under CRC development. However, recent studies estimated narrow-sense heritability and suggested that the heritability explained by known common CRC SNPs identified in GWAS was 0.65%, which is only a small fraction of the heritability explained by all common SNPs (7.42%) [16]. Additionally, though some of the variants have been validated to be associated with CRC risk by subsequent studies in Chinese Han cohorts, some of them failed to be replicated or still lack of evidence [17, 18].

These evidence suggested that many common variants associated with CRC risk, especially population specific variants were still unclear. In an effort to find additional CRC susceptibility loci, we performed a three-stage GWAS in Chinese population.

## RESULTS

### Identification of new CRC loci

**Figure 1:**
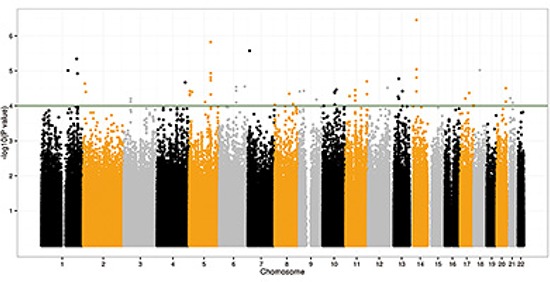
Manhattan plot of −log10 *P* values from the additive model after adjusting for age, gender and ten principal components Forty-nine SNPs were significant at the *P* < 10^−4^ level in the CRC discovery GWA scan.

A total of 1,898 subjects (932 CRC cases and 966 controls from Beijing) (Supplementary Table S1) with 1,129,636 autosomal Single-nucleotide polymorphisms (SNPs) were qualified for further GWAS analyses after QC. A low inflation factor (λ = 1.018) implied a low possibility of significant hidden population substructure between cases and controls. *P* values presented in Figure 1 are derived from the additive model in the logistic regression analyses. A total of 51 SNPs were selected (49 new targets and 2 previous hits, see Methods) for genotyping in Stage 2 including an independent sample of 1,759 cases and 1,875 controls from Jiangsu Province (Supplementary Table S1 and S2). 7 SNPs exhibited a *P* value lower than 0.05 were further assessed in Stage 3 including additional 943 cases and 1,838 controls from Beijing (Supplementary Table S2). In Stage 3, rs12522693 and rs10035791 at 5q23.3, rs17836917 at 17q12, rs6983267 at 8q24.21 and rs10795668 at 10p14 remained to be significantly associated with risk of CRC, consistent with those observed in Stage 1 and 2 (Table 1; Supplementary Table S3). In a meta-analysis of three stages, two loci (rs12522693 at 5q23.3, OR = 1.31, *P* = 2.08 × 10^−8^; and rs17836917 at 17q12, OR = 0.75, *P* = 4.55 × 10^−8^) showed evidence of association, which was statistically significant after adjustment for multiple testing (*P* < 5 × 10^−8^) (Table 1).

**Table 1:** Summary of GWA scan and replication studies for 4 SNPs consistent in three stages

SNP	Study	Cases^b^	Controls^b^	MAF^c^		OR_add_	*P*_add_	*P*–Q^d^
				Cases	Controls	(95% CI)		
rs12522693	GWAS	16/242/673	13/172/780	0.15	0.10	1.55(1.27–1.89)	1.86 × 10^−5^	
5q23.3	Replication I	34/404/1321	21/367/1487	0.13	0.11	1.27(1.10–1.46)	1.10 × 10^−3^	
G/A^a^	Replication II	16/212/715	18/360/1460	0.13	0.11	1.23(1.04–1.46)	1.61 × 10^−2^	
	Combined All					1.31(1.19–1.45)	2.08 × 10^−8^	0.19
rs10035791	GWAS	23/272/635	17/208/740	0.17	0.13	1.51(1.25–1.82)	1.54 × 10^−5^	
5q23.3	Replication I	42/463/1249	33/442/1386	0.16	0.14	1.17(1.03–1.33)	1.98 × 10^−2^	
G/A^a^	Replication II	23/252/668	31/433/1366	0.16	0.14	1.20(1.03–1.41)	2.10 × 10^−2^	
	Combined All					1.25(1.14–1.36)	8.20 × 10^−7^	0.08
rs80007597	GWAS	13/230/688	9/162/793	0.14	0.09	1.59(1.29–1.96)	1.16 × 10^−5^	
5q23.3	Replication I	27/386/1335	19/346/1493	0.13	0.10	1.25(1.08–1.45)	2.73 × 10^−5^	
G/C^a^	Replication II	14/201/726	29/329/1475	0.12	0.11	1.17(0.98–1.39)	7.64 × 10^−2^	
	Combined All					1.29(1.17–1.42)	3.63 × 10^−7^	0.07
rs17836917	GWAS	6/130/796	21/179/765	0.08	0.11	0.64(0.51–0.80)	6.15 × 10^−5^	
17q12	Replication I	12/308/1439	34/371/1470	0.09	0.12	0.79(0.68–0.92)	1.80 × 10^−3^	
G/A^a^	Replication II	7/159/777	21/379/1438	0.09	0.11	0.78(0.65–0.94)	9.43 × 10^−3^	
	Combined All					0.75(0.68–0.83)	4.55 × 10^−8^	0.27

a Major/minor alleles

b Variant homozygote/Heterozygote/Wild type homozygote

c Minor allele frequency (MAF)

OR_add_, P_add_: calculated by additive model adjusted for age, gender and first ten PC

d *P* value of Cochran's Q test.

### Haplotype analysis at 5q23.3

At 5q23.3, the lead SNP rs12522693 was in moderate LD (linkage disequilibrium) with rs10035791 (r^2^ = 0.683) and rs80007597 (r^2^ = 0.774) in our GWAS control data. Haplotype analysis suggested that individuals with the high-risk haplotype (rs10035791A-rs12522693A-rs80007597C) had a 1.40-fold increased risk of CRC compared with individuals with the most common haplotype (GGG) (*P* = 8.44 × 10^−10^) (Supplementary Table S4).

### Imputation analysis

**Figure 2:**
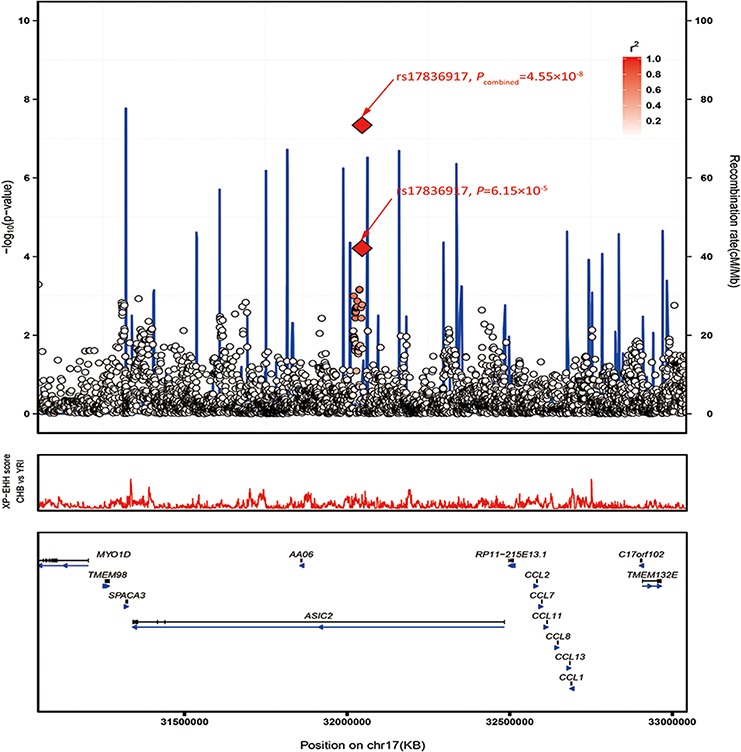

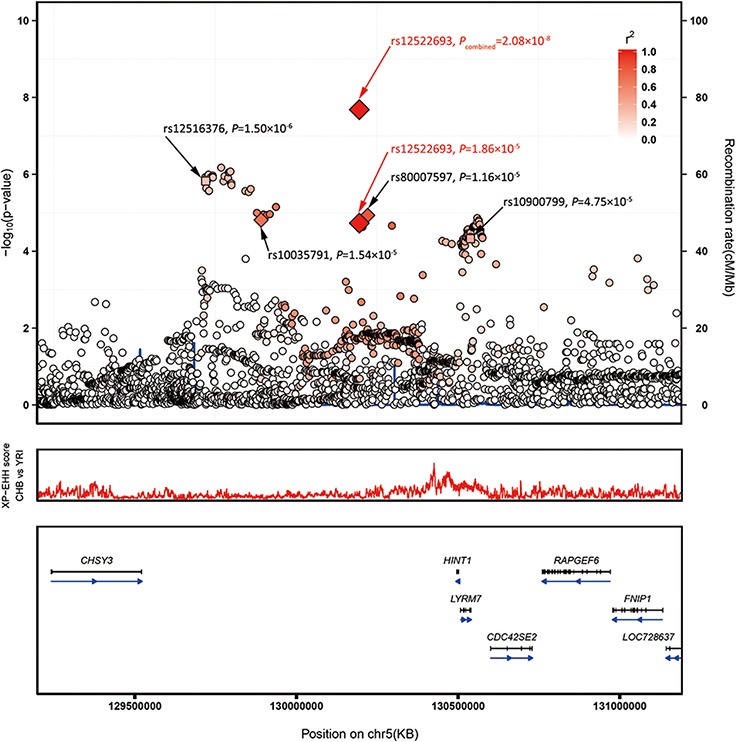
Regional plots of the two marker SNPs associated with CRC Regional plots of association results and recombination rate for (A) 5q23.3 (B) 17q12 in the GWAS discovery stage. The results (−log10P) are shown for SNPs in the region 400 kb upstream or downstream of the marker SNP. The results of successfully validated SNPs are shown as diamond and the results of SNPs failed in the validation are shown as square; the key SNPs are shown as red text and the linkage disequilibrium values (r2) for the other SNPs are indicated by the heat scale. The genes within the region of interest are annotated, and the direction of transcripts is shown by arrows. **A.** Regional plot of rs12522693 association between cases and controls in the GWAS discovery stage. Regional plot of rs17836917 association between cases and controls in the GWAS discovery stage.

Using imputation analyses based on data from the 1000 Genomes Project (Phase I integrated variant set release, v3), we tested the associations of the SNPs (imputed info >0.5, MAF >0.05) surrounding the two lead SNPs in a 400-kb window. We observed a series of significant signals around rs12522693 at 5q23.3 (*P* < 1.0 × 10^−4^) (Figure 2; Supplementary Table S5).

### Function annotation of identified SNPs

We further explored potential functional variants tagged by the two lead SNPs using data from ENCODE project (see Methods). By querying the database of 1000 Genomes, we identified 209 SNPs correlated (r^2^ > 0.5) with SNPs rs12522693 at 5q23.3 and 25 SNPs with SNPs rs17836917 at 17q12. One SNP rs12518203, which is moderately correlated with rs12522693 (r^2^ = 0.54 in Asian population), maps to region with signal of open chromatin and with DNAse hypersensitivity sites in both HCT-116 and Caco-2 cell lines. The minor allele A of rs12518203 was predicted to greatly alter the affinity of transcription factor CEBPB (PWM score from 9.1 to (−1.5). In addition, we found an eQTL (expression quantitative trait loci) signal (T-cells) in rs10035791 [19], which is located in the newly identified haplotype. The minor allele of the SNP can significantly increase the expression of gene *CDC42SE2*.

### Nearby genes expression

We also performed differential expression analysis on genes within 1 Mb regions around the lead SNPs in two public databases (Supplementary Table S6A). Most Genes (15/20) were differently expressed in tumour tissues compared with adjacent normal in at least one database. At 5q23.3, only two genes were detected (*HINT1* and *RAPGEF6*) in microarray data and *HINT1* was significantly down-regulated in tumor tissues; and in TCGA database, three nearby genes (*LYRM7*, *CDC42SE2*, and *FNIP1*) were significantly down-regulated in tumor tissues but *CHSY3* was significantly up-regulated. At 17q12, the expression of ASIC2 gene was relatively low and did not show any significant difference between tumor and non-tumor tissues, but three genes (*MYO1D*, *CCL8*, and *CCL13*) nearby shared consistent patterns of differential expression in both databases. Further eQTL analysis performed in the colon tumor tissues from the TCGA project suggested that rs17836917 was significantly associated with the expression of CCL family genes nearby (Supplementary Table S6B).

### MAF difference of the lead SNPs

We observed that both SNPs rs12522693 and rs17836917 showed slight minor allele frequency difference between different ancestries. For rs12522693, the A allele is 16% in Caucasian Europeans, which is slightly higher than the frequency among Chinese (1000 Genomes, Phase I integrated variant set release, v3). Furthermore, a bunch of positive selection signal was observed in the nearby region (Figure 2A). SNP rs17836917 is rare in European (MAF = 0.03) and African (MAF = 0.02) ancestries.

## DISCUSSION

In this study, we identified two novel, genome-wide significant CRC susceptibility loci at 5q23.3 and 17q12 and validated two known loci at 8q24 and 10p14 in Chinese Han ancestry using a three stage GWAS design. 10 of 36 previously published variants were consistently associated with CRC in our Stage 1 (Supplementary Table S7). Some other published SNPs failed to replicate in our Stage 1, which may due to our modest sample size or differences in LD structure between different ancestries.

For 5q23.3 (Figure 2A), the identified haplotype is approximate 300 kb downstream of histidine triad nucleotide binding protein 1 (*HINT1*) and 200 kb downstream of chondroitin sulfate synthase 3 (*CHSY3*). Differential expression analysis suggested that most genes in this region were differently expressed in CRC tumor compared with adjacent non-tumor tissues, indicating the important role of this region in the process of tumorigenesis (Supplementary Table S6). Recent positive selection signal based on haplotype-based tests provided more evidence that *HINT1-CDC42SE2* region may have putative function. The *HINT1* protein, a member of the histidine triad family, is highly conserved in diverse species [20]. Similar to the famous tumor-suppressor protein *FHIT*, *HINT1* was reported to be involved in tumorigenesis through participating in several important pathways (WNT, apoptosis, and DNA repair pathway etc.) [21–23]. *CHSY3* is a glycosyltransferase that has both glucuronyltransferase and N-acetylgalactosaminyltransferase activities. It has been reported that co-expression of *CHSY3* with *CHPF* confers a chondroitin sulfate polymerization activity, and chondroitin sulfate plays an important role in cancer biology [24]. *CHSY3* is generally expressed at insignificant levels, but is higher in colorectal tumor cells compared to healthy tissues [25]. Intriguingly, an eQTL study demonstrated that rs10035791 influenced the expression of cell division cycle 42 small effector 2 (*CDC42SE2*) in T cells [19], and a number of SNPs (moderately correlated with rs12522693) have been reported to be associated with the expression of *CDC42SE2* in lymphoblastoid cell lines and monocytes [26, 27]. The association provided another potential mechanism underlying the haplotype. CDC42SE2 is a cell division cycle 42 (CDC42) binding protein and plays a role in immunological action involving CDC42 [15840583]. CDC42 is a Ras-related GTP-binding protein, which is implicated in regulation of cell morphology, motility, cell cycle progression and induction of malignant transformation [28]. Additionally, *CDC42* has been reported to be highly expressed in colorectal adenocarcinoma and downregulate inhibitor of DNA binding 4 (ID4) through an epigenetic mechanism [29]. Thus, rs10035791 may take part in the process of T cells related inflammation, which was a well-known hallmark of cancer. We also performed eQTL analysis on rs10035791 and nearby genes in the colon tumor tissues but did not find any significant signal, which provide further support that rs10035791 may play a role in T cells instead of in colon tissues.

Another variant of interest, rs17836917, is located in the intron of acid-sensing (proton-gated) ion channel 2 (*ASIC2*) at 17q12 (Figure 2B). This gene encodes a member of the degenerin/epithelial sodium channel superfamily. Researchers have found that the expression of *ASIC2* is closely related with algogenesia and inflammation [30–32]. Some studies also showed that *ASIC2* is detected in intestine of adult zebrafish [33] and acid-sensing (proton-gated) ion channel 3 (*ASIC3*) is detected in inflamed human intestine [34]. But the correlations between *ASIC2* and CRC risk are currently unknown. It was worth to note that expression of *ASIC2* was relatively low in colon, so it might not be the casual gene. SNP rs17836917 lies about 500Kb upstream of a cluster of CC chemokine family genes (*CCL2*, *CCL7*, *CCL11*, *CCL8*, *CCL13*, and *CCL1*). eQTL analysis suggested that rs17836917 significantly down-regulated some of these genes (Supplementary Table S6), providing new potential explanation of the SNP's effect on CRC. Chemokines and their receptors have been identified as mediators of chronic inflammation [35–37], and chronic inflammation predisposes the formation of the preneoplastic foci and subsequently promotes tumor development and metastasis. *CCL2*, the mostly studied CC family gene, was concluded to be a crucial mediator of the initiation and progression of chronic colitis-associated colon carcinogenesis [38]. Other CC family genes mentioned above exert a similar effect on attracting monocytes, NK cells, and immature B cells by interaction with cell surface chemokine receptors [39]. This group of genes, represented by *CCL2*, have been reported to have the ability to recruit the tumor associated macrophage and to promote the progression of multiple cancers, including colorectal cancer [40–42]. Besides, we noticed that rs17836917 lack highly linked SNPs (r^2^ > 0.8) in the nearby region (Figure 2B) and the frequency of the minor allele was extremely low in the European population (MAF_European_ = 0.027, MAF_Chinese_ = 0.110), suggesting the association may be Chinese-specific. These features may be the reason why the locus was not identified in the previous GWAS studies.

In summary, we identified two novel susceptible loci (5q23.3 and 17q12) that contributed to risk of CRC in Han Chinese and validated two reported from European populations (8q24 and 10p14) and two from Eastern-Asian populations (12p13.32 and 20p12.3). We performed comprehensive bioinformatics prediction on newly identified SNPs and established multiple hypotheses to explain the underlying mechanism of the SNPs. Future studies are warranted to investigate in these CRC susceptibility loci to identify causal variants based on our work.

## MATERIALS AND METHODS

### Subjects

We performed a three-stage GWAS in a Chinese population, including a total of 3,634 cases and 4,679 controls. A summary of all cases and controls in the study is provided in Supplementary Table S1. The GWA scan phase (Stage 1) comprised 932 CRC patients and 966 controls from Beijing, followed by two stages of validation (Stage 2: 1,759 cases and 1,875 controls from Jiangsu Province, Stage 3: 943 cases and 1,838 controls from Beijing). All CRC cases were recruited in local hospitals and had pathologically proven disease. Cancer-free control subjects were recruited in local hospitals for individuals receiving routine physical examinations or in the communities for those participating screening of non-communicable diseases. In Stage 1, the cases and controls were frequency-matched by age, and in following validation stages, the cases and controls were frequency-matched by both age and gender. Subjects in Jiangsu Province were used in previously published association studies [43, 44]. At recruitment, informed consent was obtained from each subject, and this study was approved by the institutional review boards of each participating institution and medical ethics committee of Peking University People's Hospital.

### Genotyping and quality control in the GWAS

A total of 1,280,786 SNPs were genotyped in the GWA scan on 973 cases and 1,006 controls using Affymetrix Axiom Genome-Wide CHB1 and CHB2 arrays. A systematic quality control (QC) procedure was conducted on the raw genotyping data to filter both unqualified SNPs and samples (Supplementary Figure S1). We excluded SNPs if they: (1) did not map to autosomal chromosomes; (2) had call rate of <95%; (3) had minor allele frequency (MAF) <0.02; (4) deviated from Hardy-Weinberg equilibrium (HWE) (*P* < 1.0 × 10^−5^); (5) showed significantly different missing rate between cases and controls (*P* < 1.0 × 10^−5^). We removed individuals if they: (1) had overall successful genotyping call rate <95%; (2) had gender discrepancies between survey records and genetically inferred data; (3) had outlying autosomal heterozygosity rates (>6 SDs from the mean); (4) had unexpected duplicates or probable relatives (all PI_HAT >0.25). We detected population outliers using a method based on principal component analysis [45]. A set of 27,193 common autosomal SNPs (MAF >0.25) with a low LD (r^2^ < 0.05) were employed to identify population outliers in the samples passed quality control, with the founders of the HapMap (http://hapmap.ncbi.nlm.nih.gov/) trios of Yoruba in Ibadan (YRI; *N* = 90), Utah residents of northern and western European ancestry (CEU, *N* = 90), CHB (*N* = 45), and JPT (*N* = 44) as the internal controls (Supplementary Figure S2A). The PCA showed that the cases and controls were genetically matched (Supplementary Figure S2B) and the genomic control inflation factor (λ) was 1.018. After the QC process, a total of 932 cases and 966 controls with 1,129,636 SNPs were included for further analyses.

### SNP selection and genotyping in the replication study

We used the following criteria to select SNPs for further validation: (1) *P*_trend_ ≤ 1.0 × 10^−4^; (2) clear genotyping clusters; (3) SNP with the lowest *P* value when multiple SNPs in strong linkage disequilibrium (LD) (r^2^ ≥ 0.8) (Supplementary Table S8). SNPs identified in previous GWASs in Europeans with *P* < 0.05 or highly correlated SNPs with *P* < 0.05 in GWAS stage were also included (rs6983267 and rs10795668). As a result, 51 SNPs remained in Stage 2 (Supplementary Table S2). SNPs showed significant (*P* < 0.05) associations with CRC risk in Stage 2 were further selected for validation in Stage 3. Genotyping for Stages 2 and 3 was performed using the iPLEX MassARRAY platform (Sequenom, Inc, San Diego, CA). The primers and probes are available upon request. The laboratory technicians were blinded to case-control status of samples in this study.

### Functional annotation

To investigate the underlying mechanism of the lead SNPs identified in our studies, we integrated chromatin biofeature annotations with 1000 Genomes genotyping data using R Bioconductor package FunciSNP [46]. We employed DNase-seq and Chip-seq peaks of colorectal cancer cell lines from the ENCODE projects to filter the correlated SNPs lying within putative regulatory elements with Gene Expression Omnibus (GEO) accession IDs (GSM736600, GSM736493, GSM736500, GSM736587, GSM945304, GSM945162, GSM945203, GSM945206, GSM945162, GSM935426, GSM782123, GSM749748, GSM749689). Correlated SNPs (r^2^ > 0.5) within a window size of 1 Mb around the newly identified SNP were used for a FunciSNP analysis. HaploReg V2 (http://www.broadinstitute.org/mammals/haploreg/haploreg.php) was used to predict regulatory motif alteration. We performed eQTL annotation based on RegulomDB (http://www.regulomedb.org/) and eQTL browser from Chicago University (http://eqtl.uchicago.edu/cgi-bin/gbrowse/eqtl/).

### Differential expression analysis (DE analysis) and eQTL analysis

We employed two public databases to assess differential expression of genes within a window size of 1 Mb around the newly identified SNPs: (1) GSE44861 included gene expression data of 56 tumor tissues and 55 adjacent noncancerous tissues [47]. We downloaded files containing log2 normalized expression values for DE analysis. (2) Level 3 RNA-seq v2 data in The Cancer Genome Atlas (TCGA, https://tcga-data.nci.nih.gov/tcga/) included expression information of 26 paired colon adenocarcinoma and adjacent normal tissue. RSEM normalized [48] read counts were extracted for further DE analysis.

For eQTL analysis, we included 249 colon tumor tissues from TCGA with both expression profile and matched genotype data. RSEM normalized [48] read counts were extracted for further anslysis. A robust Kendall Rank Correlation Coefficient was applied to estimate the correlation between the expression of genes and the SNPs.

### Statistical analysis

We used PLINK version 1.07 [49] using logistic regression (1 degree of freedom) in additive model adjusted for age, gender and the first ten PCs if appropriate). Wilcoxon test was applied for differential expression analysis. A meta-analysis-based method was applied to combine the results from different cohorts with R package meta. A fixed-effect model was used when there was no indication of heterogeneity (*P* for Cochran's Q statistic > 0.05); otherwise, a random-effect model was adopted. Population structure was evaluated by principal component analysis in the software package EIGENSTRAT 4.2 [50]. Manhattan plot of −log_10_ *P* and chromosome region association plot was generated using the ggplot2 package [51] in R version 2.15.1. We used another R package haplo.stats to do haplotype analysis. Ungenotyped SNPs were imputed in the GWAS discovery samples using shapeit v2 (http://www.shapeit.fr/, Phasing step) and IMPUTE2 (http://mathgen.stats.ox.ac.uk/impute/impute_v2.html, Imputation step) software using haplotype information from the 1000 genomes project (http://www.1000genomes.org/, Phase I integrated variant set release, v3, across all 1 092 individuals) in the genome-wide scale. Selection signal in region association plot was based on XPEHH rank score downloaded from 1000 genome browser (http://hsb.upf.edu/). All other analyses were performed using R 2.15.1 (http://www.r-project.org./).

## SUPPLEMENTARY FIGURES AND TABLES










