# Supplementary material for: Genome-wide association study identifies two new susceptibility loci for colorectal cancer at 5q23.3 and 17q12 in Han Chinese
Source: Oncotarget. 2015 Oct 26;6(37):40327–36. doi: 10.18632/oncotarget.5530 (PMC4741898; doi:10.18632/oncotarget.5530)
Supplement: Supplementary file 1 [file oncotarget-06-40327-s001.pdf]

SUPPLEMENTARY FIGURES AND TABLES

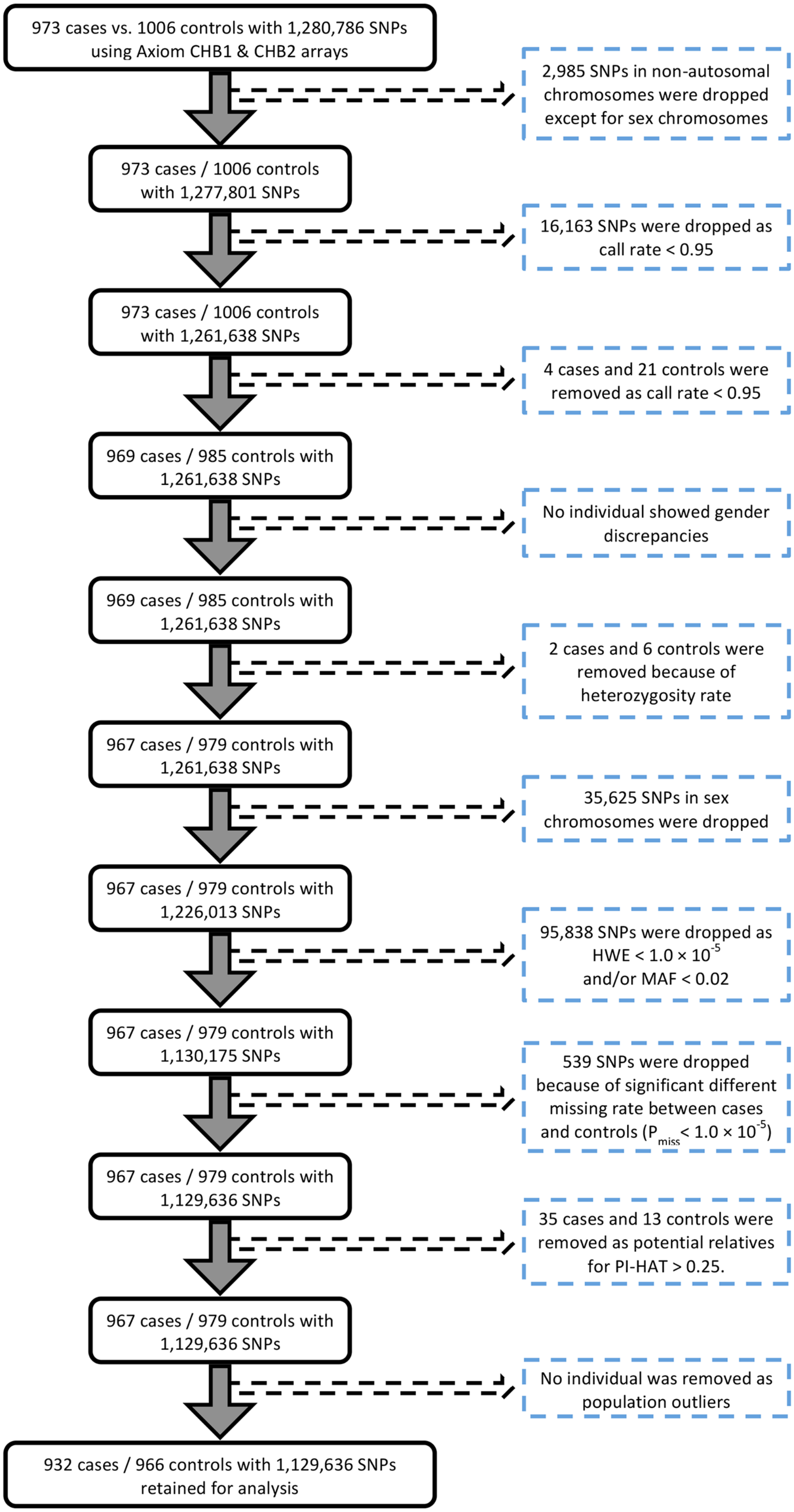

Supplementary Figure S1: A flow diagram showing the quality control process of the GWA scan.

(A) Our samples and the HapMap Phase II data

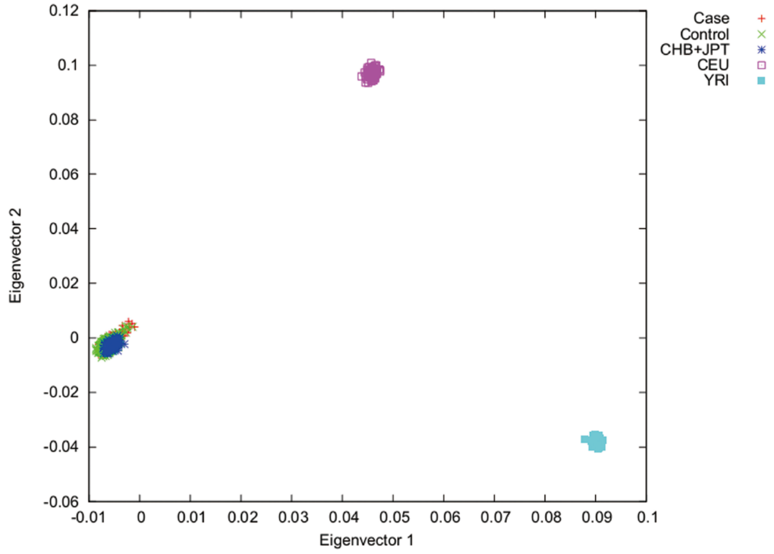

(B) Our samples

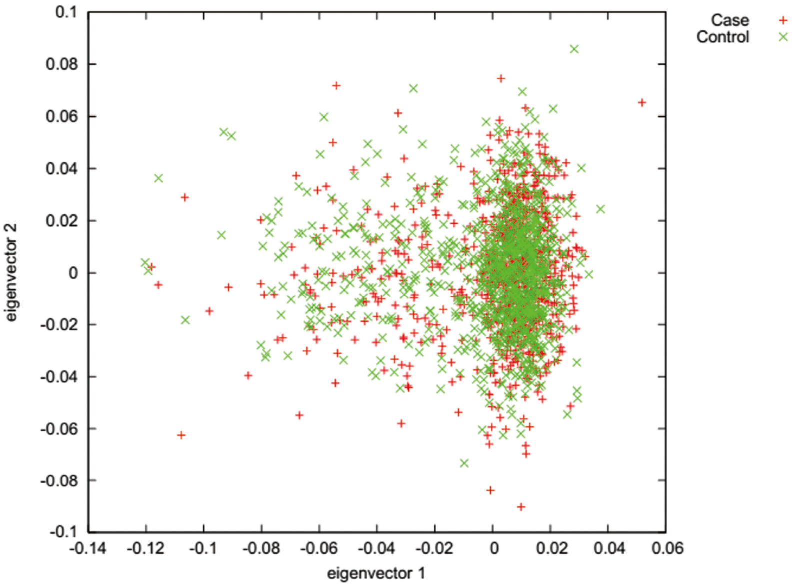

Supplementary Figure S2: Principal component analysis showing minimal evidence of population stratification.

Supplementary Table S1: Summary description of the subjects used in this study

Variables	GWA scan		Replication I		Replication II	
	case(932)	control(966)	case(1759)	control(1875)	case(943)	control(1838)
Age	61.76 ± 13.75	60.98 ± 12.91	60.16 ± 12.75	60.04 ± 13.05	59.46 ± 12.71	59.20 ± 15.26
Sex						
Male	547(58.7%)	490(50.7%)	1069(60.8%)	1119(59.7%)	572(60.7%)	1114(60.6%)
Female	385(41.3%)	476(49.3%)	690(39.2%)	756(40.3%)	371(39.3%)	724(39.4%)

Supplementary Table S2: Associations of the 51 SNPs with CRC in the GWA scan and the phase I validation

SNP	CHR	BP	Study	MAF		OR[b]	L95[b]	U95[b]	*P*[b]
				Case	Control				
rs3761685	1	160084643	GWAS	0.17	0.12	1.52	1.26	1.84	9.78E-06
T > C[a]	1	160084643	Replication I	0.14	0.15	0.95	0.83	1.09	4.63E-01
rs117979405	1	211643475	GWAS	0.07	0.04	2.02	1.50	2.73	4.53E-06
C > T[a]	1	211643475	Replication I	0.05	0.05	1.03	0.83	1.28	7.61E-01
rs3767255	1	215781574	GWAS	0.48	0.41	1.34	1.18	1.53	1.20E-05
T > C[a]	1	215781574	Replication I	0.46	0.48	0.92	0.84	1.00	5.94E-02
rs117071032	2	10885819	GWAS	0.04	0.08	0.54	0.41	0.72	2.32E-05
T > C[a]	2	10885819	Replication I	0.06	0.07	0.93	0.77	1.13	4.87E-01
rs10195641	2	16740367	GWAS	0.28	0.22	1.38	1.18	1.60	4.00E-05
T > C[a]	2	16740367	Replication I	0.25	0.25	1.03	0.93	1.14	6.04E-01
rs13063805	3	41021433	GWAS	0.33	0.27	1.33	1.15	1.53	7.55E-05
A > G[a]	3	41021433	Replication I	0.29	0.28	1.04	0.94	1.16	3.97E-01
rs117446021	3	41939699	GWAS	0.05	0.08	0.57	0.44	0.75	6.23E-05
C > A[a]	3	41939699	Replication I	0.06	0.06	0.93	0.76	1.13	4.50E-01
rs66759638	4	169417371	GWAS	0.11	0.16	0.66	0.54	0.80	2.15E-05
T > C[a]	4	169417371	Replication I	0.14	0.15	0.92	0.81	1.05	2.07E-01
rs413665	5	5609639	GWAS	0.03	0.06	0.51	0.37	0.71	3.78E-05
A > G[a]	5	5609639	Replication I	0.04	0.05	0.84	0.67	1.05	1.24E-01
rs11948748	5	18853732	GWAS	0.06	0.03	1.98	1.43	2.74	3.99E-05
G > T[a]	5	18853732	Replication I	0.04	0.04	0.96	0.76	1.22	7.60E-01
rs31015	5	96441576	GWAS	0.18	0.14	1.43	1.20	1.71	7.76E-05
G > A[a]	5	96441576	Replication I	0.15	0.17	0.92	0.81	1.04	1.68E-01
rs12516376	5	129720170	GWAS	0.19	0.14	1.56	1.30	1.87	1.50E-06
T > C[a]	5	129720170	Replication I	0.16	0.16	1.03	0.91	1.17	6.49E-01
rs10035791	5	129891761	GWAS	0.17	0.13	1.51	1.25	1.82	1.54E-05
G > A[a]	5	129891761	Replication I	0.16	0.14	1.17	1.03	1.33	1.98E-02*
rs12522693	5	130195731	GWAS	0.15	0.10	1.55	1.27	1.89	1.86E-05

(*Continued*)

SNP	CHR	BP	Study	MAF		OR[b]	L95[b]	U95[b]	*P*[b]
				Case	Control				
G > A[a]	5	130195731	Replication I	0.13	0.11	1.27	1.10	1.46	1.10E-03*
rs80007597	5	130221632	GWAS	0.14	0.09	1.59	1.29	1.96	1.16E-05
G > C[a]	5	130221632	Replication I	0.13	0.10	1.25	1.08	1.45	2.73E-03*
rs10900799	5	130539728	GWAS	0.13	0.09	1.56	1.26	1.93	4.74E-05
A > G[a]	5	130539728	Replication I	0.12	0.11	1.12	0.97	1.29	1.18E-01
rs3778032	6	100883851	GWAS	0.38	0.44	0.75	0.66	0.86	2.88E-05
T > G[a]	6	100883851	Replication I	0.41	0.42	0.99	0.90	1.08	7.84E-01
rs9371582	6	152632823	GWAS	0.21	0.16	1.45	1.22	1.72	2.81E-05
C > T[a]	6	152632823	Replication I	0.17	0.19	0.92	0.81	1.03	1.59E-01
rs2107814	7	10280986	GWAS	0.26	0.33	0.71	0.61	0.82	2.66E-06
C > A[a]	7	10280986	Replication I	0.30	0.29	1.04	0.94	1.15	4.51E-01
rs57662641	8	1226396	GWAS	0.31	0.25	1.33	1.15	1.54	9.33E-05
T > C[a]	8	1226396	Replication I	0.27	0.28	0.97	0.87	1.08	5.69E-01
rs1458126	8	87865200	GWAS	0.22	0.17	1.41	1.19	1.66	4.54E-05
G > A[a]	8	87865200	Replication I	0.20	0.20	0.96	0.86	1.08	5.47E-01
rs76257136	8	109907339	GWAS	0.04	0.07	0.55	0.40	0.74	8.84E-05
G > A[a]	8	109907339	Replication I	0.05	0.06	0.94	0.77	1.15	5.47E-01
rs6983267	8	128413305	GWAS	0.47	0.42	1.18	1.04	1.35	1.24E-02
T > G[a]	8	128413305	Replication I	0.45	0.42	1.11	1.01	1.22	2.88E-02*
rs76250944	9	4307649	GWAS	0.11	0.07	1.65	1.30	2.10	3.87E-05
T > G[a]	9	4307649	Replication I	0.08	0.09	0.88	0.75	1.03	1.20E-01
rs77249765	9	28592212	GWAS	0.06	0.10	0.60	0.47	0.76	3.73E-05
C > T[a]	9	28592212	Replication I	0.08	0.08	0.93	0.79	1.10	3.87E-01
rs143237349	9	103647166	GWAS	0.06	0.10	0.61	0.48	0.78	6.62E-05
G > A[a]	9	103647166	Replication I	0.08	0.07	1.07	0.90	1.28	4.37E-01
rs10795668	10	8701219	GWAS	0.32	0.35	0.85	0.74	0.97	1.82E-02
G > A[a]	10	8701219	Replication I	0.34	0.38	0.88	0.80	0.97	7.30E-03*
rs3812699	10	72071648	GWAS	0.45	0.39	1.32	1.16	1.51	4.25E-05
A > G[a]	10	72071648	Replication I	0.43	0.42	1.03	0.93	1.13	5.92E-01
rs4570507	10	72153946	GWAS	0.40	0.34	1.31	1.14	1.50	9.26E-05
G > A[a]	10	72153946	Replication I	0.36	0.37	0.96	0.88	1.06	4.46E-01
rs16927648	10	72207860	GWAS	0.44	0.38	1.33	1.16	1.52	3.95E-05
A > C[a]	10	72207860	Replication I	0.39	0.39	0.98	0.89	1.07	6.21E-01
rs6858	10	81142365	GWAS	0.48	0.42	1.33	1.16	1.51	3.45E-05
A > G[a]	10	81142365	Replication I	0.42	0.44	0.94	0.85	1.03	1.57E-01
rs1605871	11	24640181	GWAS	0.25	0.31	0.74	0.65	0.86	5.30E-05

(*Continued*)

SNP	CHR	BP	Study	MAF		OR[b]	L95[b]	U95[b]	*P*[b]
				Case	Control				
T > C[a]	11	24640181	Replication I	0.28	0.28	1.00	0.91	1.11	9.55E-01
rs2509928	11	58405458	GWAS	0.35	0.41	0.75	0.66	0.86	3.54E-05
T > C[a]	11	58405458	Replication I	0.37	0.37	0.99	0.90	1.08	7.88E-01
rs80273176	11	127847209	GWAS	0.15	0.21	0.69	0.58	0.82	2.00E-05
C > T[a]	11	127847209	Replication I	0.16	0.18	0.87	0.76	0.98	2.23E-02*
rs1944853	11	128571644	GWAS	0.13	0.09	1.56	1.26	1.94	4.63E-05
C > A[a]	11	128571644	Replication I	0.11	0.10	1.07	0.92	1.25	3.76E-01
rs7974919	12	116174409	GWAS	0.46	0.39	1.32	1.16	1.51	3.05E-05
C > T[a]	12	116174409	Replication I	0.41	0.43	0.93	0.85	1.02	1.16E-01
rs10507535	13	46230860	GWAS	0.33	0.39	0.76	0.66	0.87	5.44E-05
G > A[a]	13	46230860	Replication I	0.36	0.37	0.97	0.88	1.07	5.52E-01
rs4143042	13	49493648	GWAS	0.51	0.44	1.34	1.17	1.53	1.67E-05
C > T[a]	13	49493648	Replication I	0.47	0.47	1.00	0.91	1.09	9.36E-01
rs1449582	13	49498326	GWAS	0.44	0.38	1.31	1.15	1.50	6.25E-05
T > C[a]	13	49498326	Replication I	0.41	0.42	0.94	0.85	1.03	1.68E-01
rs7319398	13	71439474	GWAS	0.46	0.53	0.76	0.66	0.87	3.80E-05
A > G[a]	13	71439474	Replication I	0.48	0.49	0.97	0.88	1.06	4.78E-01
rs12886318	14	40198925	GWAS	0.12	0.17	0.65	0.54	0.79	9.05E-06
T > C[a]	14	40198925	Replication I	0.15	0.14	1.10	0.97	1.25	1.45E-01
rs17109774	14	40215686	GWAS	0.23	0.31	0.68	0.58	0.79	3.51E-07
G > A[a]	14	40215686	Replication I	0.26	0.26	1.03	0.92	1.14	6.47E-01
rs4899424	14	40229496	GWAS	0.38	0.44	0.76	0.67	0.87	3.87E-05
G > A[a]	14	40229496	Replication I	0.39	0.40	0.95	0.86	1.04	2.84E-01
rs17836917	17	32047282	GWAS	0.08	0.11	0.64	0.51	0.80	6.15E-05
G > A[a]	17	32047282	Replication I	0.09	0.12	0.79	0.68	0.92	1.80E-03*
rs12939240	17	54707917	GWAS	0.20	0.15	1.43	1.21	1.70	4.27E-05
G > A[a]	17	54707917	Replication I	0.18	0.18	1.02	0.90	1.15	7.46E-01
rs4789777	17	80394530	GWAS	0.13	0.08	1.54	1.24	1.91	9.71E-05
T > G[a]	17	80394530	Replication I	0.10	0.10	1.01	0.87	1.19	8.62E-01
rs670850	18	37290224	GWAS	0.35	0.42	0.74	0.64	0.84	9.58E-06
G > C[a]	18	37290224	Replication I	0.38	0.37	1.01	0.92	1.11	8.14E-01
rs7266311	20	55183614	GWAS	0.10	0.14	0.65	0.53	0.79	3.13E-05
A > G[a]	20	55183614	Replication I	0.11	0.10	1.13	0.97	1.31	1.07E-01
rs16980131	20	55183732	GWAS	0.06	0.09	0.59	0.46	0.77	7.62E-05
G > A[a]	20	55183732	Replication I	0.08	0.07	1.05	0.88	1.25	6.06E-01
rs991090	21	19533935	GWAS	0.33	0.39	0.75	0.65	0.86	6.06E-05

(*Continued*)

SNP	CHR	BP	Study	MAF		OR[b]	L95[b]	U95[b]	*P*[b]
				Case	Control				
T > G[a]	21	19533935	Replication I	0.35	0.34	1.08	0.98	1.18	1.45E-01
rs79323515	21	33455653	GWAS	0.11	0.15	0.68	0.56	0.82	8.22E-05
C > T[a]	21	33455653	Replication I	0.13	0.13	1.06	0.92	1.21	4.37E-01

[a]Major/Minor alleles
[b]Adjusted by age, gender and the first ten PC for GWAS and by age and gender for replications
[*]*P* value < 0.05 and consistent with GWAS discovery phase

Supplementary Table S3: Associations of other 3 SNPs in GWA scan and two validations

SNP	Study	Cases[b]	Controls[b]	MAF[a]		OR_{add} (95% CI) [e]	P_{add}
				Cases	Controls		
rs6983267	GWAS	195/478/257	174/470/321	0.47	0.42	1.18(1.04–1.35)	1.24E-02
8q24.21	Replication I	363/849/546	322/935/612	0.45	0.42	1.11(1.01–1.22)	2.88E-02
G > A[a]	Replication II	220/470/251	333/892/604	0.48	0.43	1.26(1.13–1.41)	4.73E-05
	Combined All					1.17(1.10–1.25)	7.17E-07
rs10795668	GWAS	100/388/440	137/405/415	0.32	0.35	0.85(0.74–0.97)	1.82E-02
10p14	Replication I	227/758/774	279/839/743	0.34	0.38	0.88(0.80–0.97)	7.30E-03
T > G[a]	Replication II	94/402/447	239/821/771	0.31	0.35	0.83(0.74–0.93)	1.90E-03
	Combined All					0.86(0.80–0.91)	2.96E-06
rs80273176	GWAS	19/243/650	41/308/591	0.15	0.21	0.69(0.58–0.82)	2.00E-05
11q24.3	Replication I	44/464/1239	56/548/1247	0.16	0.18	0.87(0.76–0.98)	2.23E-02
C > T[a]	Replication II	28/279/633	48/547/1238	0.18	0.18	1.02(0.88–1.19)	7.80E-01
	Combined All					0.85(0.69–1.04)	1.22E-01

[a]Major/minor alleles
[b]Variant homozygote/Heterozygote/Wild type homozygote
[c]Minor allele frequency (MAF)
OR_{add}, P_{add}: calculated by additive model adjusted for age, gender and first ten PC

Supplementary Table S4: Results of haplotype association analysis on rs10035791, rs12522693 and rs80007597

Supplementary Table S5: All SNPs identified by the GWA scan or imputation analyses at 5q23.3 and 17q12 and their associations with CRC ($P < 1.0 \times 10^{-4}$)

Supplementary Table 6A: Results of differential expression analysis on genes within 1 Mb around the newly identified SNPs in two public databases

Supplementary Table S6B: Significant results (P < 0.05) of eQTL analysis on rs17836917 and the expression of *CCL2*, *CCL7*, *CCL8*, *CCL13* and *CCL1*

Supplementary Table S7: Summary of loci associated with CRC published previously and their association with CRC in our GWAS discovery phase

Published SNP	Cytoband	Related Gene(s)	P^{a}	Source	Ref
rs1035209	10q24.2	NKX2-3 - SLC25A28	5.19E-01	Imputation	11
rs10411210	19q13.11	RHPN2	2.44E-01	Imputation	5
rs10505477[c]	8q24.21	SRRM1P1 - POU5F1B	1.62E-02*	Genotype	4,11
rs10774214[b]	12p13.32	CCND2	2.54E-03*	Imputation	13
rs10795668[c]	10p14	KRT8P16 - TCEB1P3	1.82E-02*	Genotype	7
rs10849432[b]	12p13.31	CD9, PLEKHG6, TNFRSF1A	5.75E-01	Genotype	13
rs10936599	3q26.2	MYNN	6.54E-03*	Imputation	5
rs11169552	12q13.12	DIP2B - ATF1	4.44E-01	Imputation	5
rs11196172[b]	10q25.2	TCF7L2	9.59E-01	Imputation	13
rs11255841	10p14	GATA3	6.67E-03*	Imputation	11
rs11903757	2q32.3	NABP1 - SDPR	5.88E-01	Imputation	10
rs12603526[b]	17p13.3	NXN	2.29E-01	Genotype	13
rs1321311	6p21.2	SRSF3 - CDKN1A	3.42E-02*	Genotype	9
rs174537[b]	11q12.2	MYRF, FEN1, FADS1, FADS2	8.24E-01	Imputation	13
rs1800469[b]	19q13.2	TGFB1, B9D2	3.13E-01	Imputation	13
rs2423279[b]	20p12.3	HAO1	2.47E-02*	Imputation	13
rs2427308	20q13.33	LAMA5	9.38E-01	Imputation	11
rs34245511	12q13.12	DIP2B, ATF1	8.23E-01	Imputation	11
rs3802842	11q23.1	C11orf93	2.13E-01	Imputation	6
rs3824999	11q13.4	POLD3	5.20E-01	Imputation	9
rs4444235	14q22.2	RPS3AP46 - BMP4	5.66E-01	Genotype	5
rs4779584	15q13.3	SCG5, GREM1	4.29E-01	Genotype	12
rs4925386	20q13.33	LAMA5	3.17E-01	Genotype	5
rs4939827	18q21.1	SMAD7	1.33E-01	Genotype	2
rs6469656[b]	8q23.3	LINC00536 - EIF3H	2.00E-01	Imputation	13
rs647161[b]	5q31.1	PITX1	1.16E-01	Imputation	14
rs6687758[b]	1q41	DUSP10 - CICP13	3.41E-01	Imputation	5

(*Continued*)

Published SNP	Cytoband	Related Gene(s)	P^a	Source	Ref
rs6691170	1q41	DUSP10 - CICP13	2.35E-02*	Imputation	5
rs6983267[c]	8q24.21	SRRM1P1 - POU5F1B	1.24E-02*	Genotype	10
rs7014346	8q24.21	SRRM1P1 - POU5F1B	4.34E-01	Genotype	6
rs704017[b]	10q22.3	ZMIZ1, AS1	4.93E-02*	Genotype	13
rs7229639[b]	18q21.1	SMAD7	8.26E-02	Genotype	13
rs73376930	15q13.3	GREM1, SCG5	8.63E-02	Genotype	11
rs961253	20p12.3	TARDBPP1 - BMP2	1.11E-01	Genotype	5
rs9929218	16q22.1	CDH1	9.87E-01	Genotype	5

[a]*P* value of GWAS discovery phase adjusted by gender, age and first ten PC
[b]Thirteen loci identified in Eastern Asian population
[c]SNPs involved in replication I (rs10505477 was excluded from replication because of its high linkage with rs6983267 in our GWAS control data: r2=0.979)
[*]*P* value < 0.05

Supplementary Table S8: SNPs that met the criteria in GWAS discovery phases but had strong linkage disequilibrium ($r^2 > 0.8$) with replicated SNPs in the replication stage I

SNP	CHR	BP	A1[a]	OR	L95	U95	P[b]	Replicated SNP	PSNP[c]	r2
rs2438472	5	5605830	G	0.52	0.38	0.71	4.63E-05	rs413665	3.78E-05	1.00
rs2455476	5	5606066	T	0.52	0.38	0.71	4.95E-05	rs413665	3.78E-05	1.00
rs3798492	6	100888625	T	0.76	0.66	0.86	3.68E-05	rs3778032	2.88E-05	0.99
rs3778030	6	100906046	T	0.77	0.67	0.87	8.04E-05	rs3778032	2.88E-05	0.97
rs10792172	11	58318056	C	0.75	0.66	0.86	5.03E-05	rs2509928	3.54E-05	0.88
rs2509920	11	58336544	C	0.76	0.66	0.87	7.04E-05	rs2509928	3.54E-05	0.97
rs11627784	14	40229840	G	0.66	0.55	0.80	1.56E-05	rs12886318	9.05E-06	0.94

[a]Minor alleles
[b]Adjusted by age, gender and the first ten PC
[c]*P* values of replicated SNP adjusted by age, gender and the first ten PC
